# Multiantibody Strategies for HIV

**DOI:** 10.1155/2013/632893

**Published:** 2013-06-06

**Authors:** Andrew Hiatt, Larry Zeitlin, Kevin J. Whaley

**Affiliations:** Mapp Biopharmaceutical Inc., 6160 Lusk Boulevard, C104, San Diego, CA 92121, USA

## Abstract

Vaccination strategies depend entirely on the appropriate responsiveness of our immune system against particular antigens. For this active immunization to be truly effective, neutralizing antibodies (nAbs) need to efficiently counter the infectivity or propagation of the pathogen. Some viruses, including HIV, are able to take advantage of this immune response in order to evade nAbs. This review focuses on viral immune evasion strategies that result directly from a robust immune response to infection or vaccination. A rationale for multi-Ab therapy to circumvent this phenomenon is discussed. Progress in the formulation, production, and regulatory approval of monoclonal antibodies (mAbs) is presented.

## 1. Introduction

The persistence of HIV as a global epidemic has revealed our limited understanding of how immune barriers function to protect humans against disease [1]. Soon after the recognition of the HIV virus as the causative agent of AIDS, the prediction was made that a vaccine would be available for testing within two years [2]. In the intervening 30 years, the inability to create an effective protective or therapeutic vaccine can be attributed to a number of characteristics of HIV. Some of these characteristics naturally result in evasion from immune surveillance and are also utilized by other viruses [3–5]. Viral evasion in general can involve accumulation of point mutations on immune-dominant regions of surface proteins, glycosylation of functionally pivotal residues (the glycan shield) or their association with host serum components (e.g., lipoproteins) in order to mask them from the immune system, and cell-to-cell transmission. In addition, molecular mimicry, whereby the expression of proteins structurally similar to host defense proteins, can lead to viral persistence [6–9]. These strategies can in turn result in further damaging effects. The secondary consequences of molecular mimicry range from viral-induced autoimmune disease to chronic immune stimulation, for example, HCV-induced cryoglobulinemia. 

In the particular case of HIV, immune evasion results from a variety of additional strategies. The incredible sequence diversity within each HIV subtype as well as within individuals during the course of active infection represents an enormous challenge to the immune system. Furthermore, HIV attacks the very cells that are needed to mount an effective and coordinated immune response. The destruction of CD4^+^ T cells can further facilitate viral replication [10]. Additional evasion strategies involve downregulation of MHC molecules [11–13], establishment of latent viral genomes that can result in production of infectious virus perhaps years later [14], as well as very high mutation rates of the viral genome resulting in infectious viruses that the immune response does not recognize [1, 15]. 

Evasion strategies that result directly from a robust immune response include neutralization interference by nonneutralizing antibodies (non-nAbs) [3], a potential for enhancement of viral infectivity due to the presence of anti-viral Abs [16], and the propensity of our memory immune system to become overly influenced by the earliest immune response after infection or vaccination. The uncertainties in the development of robust active immunization strategies for viruses such as HIV provide the rationale for passive immunization strategies that employ multiple mAbs as a basis for both protective and therapeutic clinical modalities against a variety of viral infections. 

## 2. Interfering Nonneutralizing Abs (Non-nAbs)

The problem of non-nAb interference has been investigated in a number of viruses and represents a viral evasion strategy that needs to be addressed if the development of new vaccines is to be successful. This type of evasion strategy also suggests that passive immunization may be an alternative. In the case of HCV, broadly crossneutralizing Abs (bnAbs) are most effective when directed against highly conserved and functionally critical epitopes (e.g., the CD81-binding site) among different genotypes [17–27]. However the binding of these HCV bnAbs may be inhibited by the presence of non-nAbs that bind proximal to the critical residues [28–34]. This hypothesis is still controversial [26] but recent experiments support the existence of interfering Ab populations [35].

In the case of influenza, humoral immunity resulting in the inactivation of the receptor-binding site on HA appears to be the main mechanism of influenza neutralization [36–39]. In addition, bnAbs often inhibit the fusion of the viral envelope with the endocytic vesicle membrane [20, 40–44]. Non-nAbs, if produced in sufficient abundance, may provide a basis for viral escape from the bnAbs [45–48]. Overall, the experimental results suggest that non-nAbs that bind to epitopes of HA may interfere with the binding of nAbs to proximal neutralization epitopes.

Further evidence that prevalent non-nAbs can result in viral escape is found in severe acute respiratory syndrome coronavirus (SARS-CoV). Vaccine strategies, directed to preventing infection, have used the SARS-S viral glycoprotein as a target [49]. This strategy has proven to be problematic since vaccination for coronavirus may result in excessive and sometimes uncontrolled cellular immune responses contributing to the severity of the disease [50]. In the case of SARS-CoV, it has been reported that a nonneutralizing mAb can disrupt the neutralizing activity of mAbs that inhibit infection *in vitro* [51, 52]. Overall, the results suggest that a cocktail of nmAbs binding to different epitopes may be a valid clinical approach [53].

The cocktail strategy may be especially relevant in the case of HIV where cytotoxic T lymphocytes and neutralizing Abs have long been known to select for immune escape mutations during the course of infection [54–57]. In addition, inactivation of bnAbs by non-nAbs has been reported [58–61]. This antagonism has been proposed to be due to steric hindrance [62]. In contrast, the observation of additive reactivity involving non-nmAbs and nmAbs suggests that multi-mAb combinations can support HIV inactivation irrespective of the individual mAb neutralizing potency [59]. In all probability, however, a cocktail approach to passive immunotherapy for HIV will need to involve highly crossneutralizing mAbs [63, 64] whose affinity and epitope locations can overcome the inhibitory effects of interfering non-nAbs.

## 3. Evasion Resulting from “Original Antigenic Sin”

The human immune system has evolved to respond very quickly and effectively to infectious challenges long after the primary infection has been resolved [65–68]. This immune memory is essentially a quick response capability that avoids the much slower process of the original immune reaction that ultimately gives rise to affinity maturation and an antibody repertoire. With memory, the antibody repertoire can be brought to bear in a matter of days, rather than weeks and months [65–68]. Whereas this rapid response can be essential to preventing repeated infections, it does however have some drawbacks that have provided the opportunity for certain viruses to continually establish successful infections. This susceptibility has to do with the characteristics of the initial immune response and the subsequent inability of the memory response to adequately broaden the repertoire of antibodies in the face of an infection by a similar or mutated strain. In essence, the diversity of a secondary immune response can be compromised by the dominance of the original immune response [69–71]. 

The first description of this phenomenon was published 60 years ago and was referred to as “original antigenic sin” (OAS) [72]. After an influenza virus infection, antibody produced after re-infection or vaccination with a related strain of virus is apparently still directed against the first strain that resulted in an immune response [73]. In other words, there was a recall of the first influenza virus experienced. This phenomenon, in which the immune system commits itself to the viral variant initially present and continues to make antibodies against the image of this virus even when contemporaneous virus has effectively shed this image, has been observed after infection by a number of viruses [36, 37, 74]. What stops the immune system from continually producing high-affinity neutralizing antibodies against emergent viral variants is not entirely clear.

One potential consequence of OAS is simply a lack of an adequate immune response to mutated virus. In addition, OAS presents a risk of the elicitation of Abs that could potentially enhance disease severity by enhancing viral infection. A prime example where this mechanism has been invoked is dengue virus. In the case of dengue, Abs derived from an initial immune response may act as agents that exacerbate disease by increasing the cellular uptake of viruses, resulting in higher viremia, a phenomenon termed antibody-dependent enhancement (ADE) [38]. While ADE has been the leading theory to explain the observation of increased risk of severe disease upon a secondary infection from a heterologous serotype, recent studies in humans have called into question ADE as the principal mechanism of increased disease risk [39, 75, 76]. Additionally, modifications to antibody Fc regions that disrupt antibody interaction with Fc*γ* receptors have been shown to be effective strategies in preventing ADE-mediated lethal disease in a mouse model [77].

In spite of the apparent drawbacks of OAS, it has been shown that individuals can mount immune responses to an HIV infection that have all the hallmarks of an OAS response and nonetheless manage to generate bnAbs that coevolve with the mutating virus. A recent study followed this evolution in a single infected individual over a three year period [78]. In spite of the propensity for matured bnAbs to maintain neutralizing activity against the founder virus, potential viral escape mutations in the vicinity of the bnAb epitope were nonetheless neutralized due to bnAbs gaining neutralization breadth during affinity maturation. OAS therefore is a complex immune response that can result in production of effective neutralizing Abs in some cases.

## 4. Repertoire Freeze and Anti-Idiotypes

One explanation for OAS is that early induction of Ag-specific B cells and consequent free Abs are able to recognize viral escape mutants with sufficient affinity to successfully compete for viral antigens and minimize the effectiveness of naïve B cells encountering the viral escape [79]. Since these previously activated B cells and antigen-specific Abs are far more abundant than the naïve B cells, they can be selected to undergo somatic hypermutation and affinity maturation that, in some cases, can drive viral escape. The benefit of this phenomenon has been proposed to reside in an adaptive immune response that limits ineffective or even pathological antibodies along a narrow idiotypic axis, hence conserving idiotypic space for functional antibody responses [74]. 

It has been observed that those Abs derived from early infection very often carry a common idiotype, termed 1F7, that has been proposed as a potential target for therapeutic anti-idiotypic suppression [74, 79]. Whereas suppression of the 1F7-bearing population can allow for a higher titre of Abs capable of neutralizing the autologous contemporaneous viruses, some evidence suggests that bnAbs can develop within the 1F7 repertoire. It has been suggested that the continual selection of the 1F7-idiotype Abs may in fact drive the V region mutations that are the hallmark of HIV bnMAbs. Six well-characterized bnMAbs (b12, VRC01, 2F5, 4E10, 2G12, and Z13e1), and perhaps others, all express the 1F7 idiotype. In addition, the 1F7 idiotype has been found in Abs derived from other chronic infections such as HCV and SIV [74].

Some potential methods for avoiding OAS have been described [37]. These include masking gp120 epitopes [80, 81], using cytokines [82], and suppressing dominant B and T cell clones [80, 83]. 

## 5. Broadly Neutralizing Antibodies

The importance of conserved epitopes that are crucial to viral infection or propagation cannot be overstated. As targets of an immune response, conserved epitopes are the foundation of an antibody repertoire containing broadly neutralizing Abs. This is true for the immune response to variety of viruses. The immune response to influenza, for example, has provided insights into the difficulty of devising effective vaccine strategies [84]. This is because in influenza, as in other viruses, the best bnMAb candidates for use in therapy and prophylaxis are not directed against the major antigenic sites. Anti-influenza mAbs with broad-range neutralization activity against highly divergent isolates are generally able to interfere with the viral fusion process in the endosomic vesicle by targeting conserved epitopes at that site. These bnMAbs are poorly induced by infection or vaccination as is the case with HIV and other viral infections. The bnMAbs against influenza and other viruses have been isolated by phage display techniques [41, 85, 86] or directly from human peripheral B cells [20, 44, 87].

Although a robust initial immune response to HIV infection is a hallmark of the disease, only about 20% of infected individuals mount an immune response that contains bnAbs. In addition, neutralizing immune responses rarely contain neutralizing antibodies against all the HIV clades. Broadly neutralizing anti-HIV mAbs are rare but there has been impressive recent progress, utilizing new mAb discovery technologies that have produced a variety of bnMAbs (Table 1) [87–94]. To date, there are approximately 50 bnMAbs that represent an essential arsenal of anti-infectious agents against HIV. 

The hope that a single bnMAb will ultimately be found that will not readily select for escape mutations has persisted since the beginning of HIV antibody discovery [95]. The proposition that infectious diseases including HIV can be managed by the use of a cocktail of mAbs was suggested over ten years ago [96]. Clearly, for HIV, a cocktail of bnMAbs would stand a better chance of avoiding selection and providing protection and therapy [3]. The remainder of this paper will focus on HIV and the use of the broadly neutralizing anti-HIV mAbs that have been developed to date.

## 6. The Effectiveness of Multi-mAb Therapy

Progress towards establishing the effectiveness of a multi-mAb approach compared to single-mAb strategies has recently been reported [97, 98]. In one report [97], in order to evaluate the therapeutic potential of multiple broadly neutralizing antibodies on established HIV-1 infection, groups of humanized mice were infected with CCR5-tropic HIV-1 isolates (HIV-1_YU2_). Humanized mice were used in order to minimize production of anti-human antibodies. 

Mice were first treated using antibody monotherapy that evaluated five different broadly neutralizing antibodies. These antibodies were selected based on their neutralizing activity as well as the breadth of clades that could effectively be neutralized *in vitro*. In addition, each mAb targeted different epitopes. The serum half-lives of these mAbs ranged up to 6.3 days. In general, using monotherapy, viremia rebounded after 14–16 days with the concomitant appearance of gp120 mutations that allowed viral escape from mAb selection. Monotherapy therefore selected for viral escapes by mutation of antibody-targeted epitopes. The ability of a trimix and a penta-mix of bnMAbs to alter the course of infection was then evaluated. In contrast to monotherapy and the trimix, all of the pentamix-treated mice remained below baseline viral loads during the entire treatment course. Prolonged control of the infection was observed with the pentamix primarily due to the long serum half-life of the injected antibodies [99]. The efficacy of these antibody-based drugs may be further enhanced with modifications that extend half-life several folds [100].

Similar experiments in humanized mice and humans where multiple mAbs were evaluated for therapeutic efficacy against established infections did not reveal a significant benefit to the combination bnMAb approach [101–103]. In those experiments, the broadly neutralizing antibodies (b12, 2G12, and 2F5 in mice; 2G12, 2F5, and 4E10 in humans) were less potent than VRC01 or the bnMAbs used in the Klein et. al. study [97]. This difference in potency as well as the inclusion of two additional mAbs to make a penta-mix may account for the different results. 

The mutli-mAb approach is similar to the combination therapies involving antiretroviral, antimicrobial, and anticancer agents since circumventing the selective pressure necessarily involves the simultaneous appearance of multiple mutations. Antibody therapy for HIV also offers the advantage of being able to specifically neutralize the virus, and can recruit other components of the immune system resulting in viral clearance from infected cells by eliciting effector functions [104]. Moreover, immune complexes from bnMAbs may augment native immunity and have far longer half-lives than antiretroviral drugs [105]. 

## 7. Multi-mAb Prevention of Transmission

A multi-mAb microbicide has demonstrated 100% efficacy in a humanized mouse model [106]. Broadly neutralizing HIV antibodies 2F5, 2G12, and 4E10 manufactured in mammalian cells and combined as MabGel have completed early clinical trials as a vaginal microbicide [107]. A Nicotiana-manufactured (see Section 9 below) multi-mAb consisting of VRC01-N, 10E8-N, and HSV8-N as an HSV/HIV microbicide is currently in development (Mapp Biopharmaceutical, 2013). Nicotiana-manufactured 2G12 mAb that was vaginally delivered has completed a small clinical trial; no product-related adverse events were reported (Julian Ma, personal communication).

Since intracellular virus would be better protected than free virus from adverse effects of antiviral factors in the genital environment such as antiviral antibodies [108], and cell-cell transmission enables HIV-1 to evade inhibition by potent CD4bs directed antibodies [109], anti-cell mAbs [110, 111] will be an important component of a multi-mAb microbicide. 

## 8. Regulatory Challenges of Multi-mAb Therapeutics

The regulatory and manufacturing challenges of a multi-mAb strategy have until recently been assumed to be nearly insurmountable. However both the regulatory and manufacturing procedures have been shown to be amenable to straightforward approaches involving FDA guidance and technological advances that have allowed for reproducible batch-to-batch potency as well as genetic stability and consistency [112]. A Phase 1 clinical trial has been performed with a three-mAb cocktail for botulinum toxin being developed by Xoma [113], and Phase 2 trials have been performed by Symphogen involving a 25-mAb and a two-mAb cocktail [114] and by Crucell (two-mAb rabies cocktail [115]).

In one recent report [112], product-specific methods addressing the polyclonality of a multi-mAb product were focused at the genetic level using a T-RFLP methodology, as well as at the protein level using CIEX- and MS-based methodologies to verify the consistency of manufactured batches. At the level of antigen reactivity, methods have been established to verify the potency of each antibody contained in each batch of the product. In December 2010, FDA published a draft Guidance for Industry entitled “Co-development of Two or More Unmarketed Investigational Drugs for use in Combination” (http://www.fda.gov/Drugs/GuidanceComplianceRegulatoryInformation/Guidances/default.htm). The recommendations in this, and an earlier draft guidance (FDA Points to Consider, February 28, 1997), may direct the development of recombinant antibody mixtures for multidisease products (e.g., HSV/HIV microbicides). New and cost-efficient cell banking and manufacturing concepts for multi-mAb products have been described [112, 115–121], and it has been demonstrated that a complex mAb composition containing 25 antibodies can be manufactured in a highly consistent manner in a scaled-up production process. This single-batch manufacturing concept represents a relatively simple approach to the production of complex mixtures of antibodies with an integrated high flexibility with respect to number of antibodies and design of composition.

## 9. Alternative Production Systems

Given the enormity of the HIV problem as well as the cost sensitivity inherent in the economic environments where HIV therapies are most urgently needed, alternatives to the mammalian cell culture technology might be appropriate. In the past, cost of production for life-threatening antibody-based drugs has not been a significant factor in determining the price of any particular drug [122]. In the case of HIV however the shear size of the unmet need may be beyond the current worldwide manufacturing capability of animal-cell-based production [122]. 

The cell culture system reported by Frandsen et al. [112] employed a recombination target site for integration of each individual mAb into the same genomic site thereby minimizing genomic position effects caused by the expression cassettes [123]. Each of these mammalian production cell lines is expected to be similar with regard to growth and production characteristics. Other production systems however obviate the need for uniform genetic integration events since no genomic integration is involved in generating the antibody producing cells. For example, using a transient plant system, expression of each mAb can result from the infection of plant cells by *Agrobacterium tumefaciens* [124]. This infection is performed after introducing several provectors into the *Agrobacterium *that can deliver the viral components and the foreign genes to plant cells. In this sense, *Agrobacterium *is the vehicle for primary infection and systemic movement in the plant, whereas the ultimately recombined, functional viral replicon provides cell-to-cell spread, amplification, and high expression. None of the provectors contain plant-selectable markers (e.g., kanamycin resistance), and they are not selected for genome integration and expression (a process that can consume years). Instead, the *Agrobacterium*-delivered provectors are engineered with specific recombinase sites that, when codelivered into the cell with their counterpart enzyme (phage C31 integrase), recombine efficiently *in planta*, forming the completed viral replicon. The mixing and codelivery of multiple *Agrobacterium*-based vectors, each containing a separate component of the viral replicon, is a fast and efficient method for expressing a wide range of proteins combining different elements. The combinatorial and iterative nature of antibody research is well matched to such an approach [124].

Unlike traditional transgenic plant production of mAbs which requires from months to years for scale-up (Table 2), the transient expression technology has proved not only versatile, but capable of rapid, high-yielding production of a variety of proteins [125]. Its ability to rapidly produce gram quantities of mAb within 10 days (from vector delivery to purified mAb) is exceptional in biopharmaceutical manufacturing. Dozens of mAbs to multiple pathogens have been produced in this fashion, and to date, all have been similar to those produced in mammalian cell culture when analyzed by a variety of *in vitro* and *in vivo* assays. In economic terms, the costs of manufacturing of mAbs for preclinical development using traditional mammalian cell culture (e.g., CHO or NS0) can be cost-prohibitive—cGMP—production of a mAb from CHO or NS0 as a contract manufacturer would cost a minimum of $5 M [122]. In contrast, production in the plant transient system under GMP has been estimated to require approximately one-sixth of that cost. It is also anticipated that significant cost-savings in the final commercial product will be realized where it is estimated that the drug substance at commercial scale will cost less than $50/g [126]. 

Glycosylation has historically been the only practical difference between mAbs produced in mammalian cell culture and in plant tissue [127]. Because of the potential for plant glycans to affect pharmacokinetics as well as immunogenicity in humans, a transgenic *Nicotiana benthamiana *line with xylosyltransferase and fucosyltransferase activity effectively knockedout has been frequently used. The resulting glycans in the double-knockout are more homogeneous than current FDA-approved mAbs produced in mammalian cell culture. The 2G12 mAb (Table 1), when produced in the double-knockout plants to yield glycans without xylose or fucose, showed significantly enhanced binding to Fc*γ*RIIIa and mediated higher antiviral activity [128]. It is noteworthy that although non-fucosylated mAbs are rare in CHO- and NS0-derived mAb products in comparison to the plant-produced mAb, a large fraction (~30%) of human serum IgG is non-fucosylated [129]. It is particularly relevant for *in vivo* studies that plant-derived mAbs have serum pharmacokinetics identical to those of mAbs produced in mammalian cell culture [130].

## 10. Summary

Viruses can escape the mammalian immune system by a variety of methods. The evasion methods that derive directly from the characteristic of our immune response include interfering non-nAbs, antibody-dependent enhancement of infection, and an attenuation of the immune response resulting in a limited diversity of Abs to mutated virus. There is a compelling rationale for multi-mAb products that can serve as both preventive and therapeutic drugs for HIV in particular and potentially for a variety of other infections that have proven to be recalcitrant to vaccine development. The availability of numerous broadly neutralizing mAbs for HIV provides the impetus for determining the most appropriate mAb combinations. In the future, multi-Ab candidates for HIV (and other viruses) may use a transformative strategy of epitope delineation based on neutralization fingerprints for screening sera or characterizing antibody specificities induced upon infection or vaccination [131]. In addition, new scalable production systems as well as a favorable regulatory environment may enable multi-mAb products for infectious diseases to be commercialized.

## Figures and Tables

**Table 1:** Broadly neutralizing monoclonal antibodies (bnMAbs) against HIV.

Epitope	bnMAb	Discovery method	Median or range of IC_50_ values (*μ*g mL^−1^)	References
MPER^1^	2F5	EBV tfm^2^	3.8–7.8 [132]	[133]
	4E10	EBV tfm^2^	3.4 [134]	[88]
	10E8, 7H6	Neutralization assays^6^	0.3–1.5 [135]	[135]
	Z13e1	Phage display	—	[57]

V1V2^3^	PG9	Neutralization assays^6^	0.1–9.4 [134]	[89]
	PG16	Neutralization assays^6^	0.1–7.6 [136]	[89]
	CH01–04	EBV tfm^2^	0.02–4.9 (CH04) [137]	[137]
	PGT141–145	Neutralization assays^6^	0.2–2.1 [134]	[134]

V3^4^	2G12	EBV tfm^2^	2.4 [a]	[91]
	PGT121–123	Neutralization assays^6^	0.03–0.05 [134]	[134]
	PGT125–131	Neutralization assays^6^	0.02–0.5 [134]	[134]
	PGT135–137	Neutralization assays^6^	0.2–7.8 [134]	[134]
	HGN194	B cell immort^10^	0.1–3.7 [138]	[138]

CD4 bs^5^	b12	Phage display	2.8 [134]	[92]
	HJ16	B cell immort^10^	0.01–9.8 [138]	[138]
	VRC01–03	RSC3^7^	0.3 (VRC01 [134])	[139]
	NIH45-46	gp120, 140 probes^8^	0.06–1.9 [140, 141]	[140]
	3BNC55, 60, 62, 117	gp120, 140 probes^8^	0.01–1.4 (BNC117 [141])	[140]
	12A12, 21, 30	gp120, 140 probes^8^	0.08–2.6 (12A12 [141])	[140]
	VRC-PGV04, 4b	RSC3^7^, pyrosequencing^9^	0.2 (PGV04 [134])	[139]
	8ANC37, 131, 134	gp120, 140 probes^8^	0.06–6.3 (131 [141])	[140]
	1B2530	gp120, 140 probes^8^	0.06–9.8 [141]	[140]
	1NC3, 7, 9	gp120, 140 probes^8^	0.02–1.2 (INC9[141])	[140]

^1^Membrane-proximal external region of gp41.

^2^EBV transformation of B cells.

^3^V1V2 site on gp120.

^4^Glycan V3 site on gp120.

^5^CD4 binding site on GP120.

^6^Neutralization assays of B cells from infected donors.

^7^Resurfaced stabilized core 3 probe.

^8^Somatic mutation primers, gp120 and gp140 probes.

^9^454 pyrosequencing to characterize additional VRC01-like antibodies from HIV-1—infected individuals.

^10^Efficient B cell immortalization and high throughput screening.

**Table 2:** Transient plant technology: the advantage of RAMP*.

Expression system	Time to mg of mAb	Time to g of mAb
Mammalian cell culture	2–6 months	3–12 months
Transgenic animals	>12 months	>12 months
Transgenic plants	12 months	>24 months
RAMP	14 days	14–20 days

*adapted from Hiatt and Pauly, 2006 [124].
